# The impact of labour market shocks on mental health: evidence from the Covid-19 first wave

**DOI:** 10.1007/s40888-023-00304-z

**Published:** 2023-05-26

**Authors:** Francesco Bogliacino, Cristiano Codagnone, Frans Folkvord, Francisco Lupiáñez-Villanueva

**Affiliations:** 1 Dipartimento di Scienze Economiche, Università Degli Studi Di Bergamo, Bergamo, Italy; 2 Dipartimento di Scienze Sociali e Politiche, Università Degli Studi Di Milano, Milan, Italy; 3 Tillburg School of Humanities and Digital Sciences, Tilburg University, Tilburg, The Netherlands; 4 Faculty of Information and Communication Science, Universitat Oberta de Catalunya, Barcelona, Spain

**Keywords:** Stress, Anxiety, Depression, Negative economic shocks, Covid-19, E24, I18, C83, I39

## Abstract

In this study, we estimate the effect of a negative labour market shock on individuals’ levels of stress, anxiety, and depression. We use a dataset collected during the first wave of the Covid-19 pandemic, on a representative sample of citizens from Italy, Spain, and the United Kingdom, interviewed on three occasions. We measure stress, anxiety and depression and labour shocks using validated scales. Our research design is a standard difference-in-differences model: we leverage the differential timing of shocks to identify the impact on mental health. In our estimations, a negative labour shock increases the measure of stress, anxiety, and depression by 16% of a standard deviation computed from the baseline.

## Introduction

Recent policy debates have featured the relationship between income losses experienced in the labour market (henceforth negative labour market shocks) and problems of toxic stress and anxiety that potentially lead to depression and other pathologies. In this article the main outcome is toxic stress, anxiety, and depression. Toxic stress occurs when the body’s regulatory capacity is overwhelmed by the level of stimuli to which it is exposed; anxiety is a reaction (usually to stress) that includes worrying, nervousness or uneasiness; and depression is a condition affecting emotions and beliefs, that determines the ability (or lack thereof) to function as an individual (Beck & Beamesderfer, 1974). Our scale measures the severity of several symptoms that can manifest of stress, emotional, and depressive disorders.

Knowing how negative labour market shocks affect mental health informs our understanding of the consequences of poverty, macroeconomic recessions and financial crisis and motivates our research. Besides having established that poverty has psychological consequences over self-control and cognitive function (Bogliacino & Montealegre, 2020; Bogliacino et al., 2021; Haushofer & Fehr, 2014; Mani et al., 2013), the scientific literature has argued that these psychological effects may also extend to mental health (Ridley et al., 2020). Nevertheless, due to the nature of the identification strategy, we learned from previous studies that poverty *alleviation* improves mental health, as positive income and not negative income shock is used as an exogenous variation.

The literature around major adverse aggregate shocks leads to similar conclusions. The 1997–1998 crisis, the Great Recession (2007–2009) and the Euro crisis (2010–2013) have shown that suicide rates are sensitive to macro-economic shocks, and particularly to unemployment (Barr et al., 2015; Branas et al., 2015; Chang et al., 2009; Economou et al., 2013; Gili et al., 2012; Reeves et al., 2012, 2014; Stuckler et al., 2009). Covid-19 further substantiated this claim. It had been predicted, for instance, that the Covid-19 pandemic would have resulted in excess suicides and deaths related to mental problems both in Canada and the US (Bianchi et al., 2020; McIntyre & Lee, 2020a, 2020b). Halting the economic system and forcing individuals to remain at home (or to have severe social restrictions) via non-pharmaceutical interventions (NPI) was expected to worsen mental health in the population (Brooks et al., 2020). This literature is mainly correlational and unclear on mechanism.

Underlying much of this policy debate is the research question: Do income losses lead to more stress, anxiety, and depression?

Addressing this question poses an empirical problem, as omitted variables or reverse causality may confound observed correlations. For instance, being under severe stress or anxiety worsens job performance and may lead to being fired. Being poor or living in an underprivileged neighbourhood may simultaneously explain why people face losses and feel anxious. The empirical literature followed two routes to circumvent the problem of self-selection: either exploit or randomly assign *income windfalls* (Angeles et al., 2019; Baird et al., 2013; Eyal & Burns, 2019; Haushofer & Shapiro, 2016; Haushofer et al., 2020; Kilburn et al., 2018) or, as the literature mentioned in the previous paragraph, exploit an *intention to treat* based on aggregate negative events. The first is predicated on the symmetry of the relationship between negative and positive shocks, the second on the plausible exogeneity of those aggregate events.

In this paper, we provide an alternative empirical strategy. We tracked the labour market status of individuals during the first wave of the Covid-19 pandemic and measured their level of stress, anxiety, and depression, providing a standard setting for a difference-in-differences research design. Our inference does not entail symmetry of income windfalls’ effect with respect to losses. Moreover, our research design provides more compelling evidence on the *labour market* as a mechanism through which psychosocial stressors lead to mental health problem. This is not the case for studies estimating the reduced form impact from aggregate shocks, where multiple channels are working simultaneously.^1^ Of course, we need to convince the reader that our setting, involving changes in labour market status related to a significant negative aggregate shock, does not suffer from similar confounds, but this is where our longitudinal dimension plays a leading role.

The current study provides compelling causal evidence that negative shocks suffered in the labour market increase the levels of stress, anxiety, and depression, as measured by a validated scale (Cardeña et al., 2000; Lovibond & Lovibond, 1995). We use a unique dataset collected during the first wave of the Covid-19 crisis, longitudinally following a sample of citizens in Italy, Spain, and the United Kingdom (Bogliacino et al., 2020). Data are collected three times during a month. We estimate the change in outcome observed in individuals exposed to negative labour shocks with respect to control. We identify the shock via the longitudinal change in the labour market (both through a loss of employment or income source) and use a standard difference-in-differences design using a two-way fixed effect estimator (TWFE). Given restricted access to hospitals and health services during the 2020 lockdown, administrative data may seriously underestimate the prevalence of mental health issues, thus access to longitudinal data, even if self-reported, is fundamentally relevant.

In our estimations, a negative labor shock increases our stress, anxiety, and depression index by 16% of a standard deviation with respect to the baseline. This result is robust to using different measures of exposure to shocks. To quantify the severity of the effect, we can translate the effect in terms of duration of the symptoms. The scale measures the duration of the symptoms over one week. One point corresponds to 36 h and the standard deviation in the baseline is 27 h. In this setting, the effect is equivalent to four additional hours of toxic stress, anxiety, and depression.

In this article, the source of variation is having experienced the shock in either the second or the third wave of data collection. To understand the validity of our research design, we proceed by steps. Our sample includes four cohorts of participants: the never treated (*C*_*0*_), those reporting a shock in the first data collection (*T*_*1*_), those reporting a shock in the second data collection (*T*_*2*_), and those reporting a shock in the third data collection (*T*_*3*_). T_1_ should be excluded, as we cannot separate their invariant characteristics from the shock, and we allow for a group-specific trend. For T_2_ and T_3_, we can identify the causal impact based on the standard assumption of parallel trend. The main threat to identification comes from other time-varying unobservables that may confound the labour market shock. Staggered treatment between *T*_*2*_ and *T*_*3*_ renders identification more plausible. Nevertheless, it also introduces the problem of heterogeneity: TWFE uses information from those already treated to estimate the counterfactual trend. A bias would come in, had the treatment be heterogeneous or dynamic. As an answer to that, we treat as separate DiD the comparisons *C*_*0*_–*T*_*2*_ and *C*_*0*_–*T*_*3*_. Following Callaway and Sant'Anna (2021), we use the never treated as a control. The results are robust.

Since this study takes place in the context of Covid-19, time-varying policies may represent potential confounds. Two reasons suggest that our results are robust: (1) during the first wave and especially during our limited period, differentiated policies were rejected in favour of NPI enforced universally; (2) since we have the information on the day on which the participant responded to the questionnaire, we can introduce day fixed effect to capture policy change. Results are robust even when we control for day-by-country fixed effects and even when we allow for dynamic effects of gender, income and education. Finally, when we look at the measurement error for the independent variable, the results do not change.

After establishing this main result, we explore its possible heterogeneity. The literature suggests the existence of relevant heterogeneity for the effect of income losses on mental health according to financial strain or physical health (Ridley et al., 2020). We collected financial strain and physical health status in the first wave. We could not find support for the presence of heterogeneity along these dimensions. We also perform equivalence tests to bound the interaction effect around zero. In our test, we exclude that the effect is larger than a predefined threshold, although the threshold is relatively large (70% of a standard deviation). For additional heterogeneity analyses, we measured a battery of preference traits, expectations, and fears towards the future, but neither the former nor the latter moderate the effect. Finally, since we gathered information on the difficulty in staying at home, we tested whether this reported difficulty systematically moderates the effect. Data are inconclusive. We do not claim causality in this section as we operate under reduced power and more restrictive assumptions.

This paper contributes to the literature on the evolution of mental health problems during major events (Barr et al., 2015; Economou et al., 2013; Gili et al., 2012; Thern et al., 2017; Wang & Fattore, 2020), by exploring the labor market channel. We interpret this effect mainly through a loss of status, as a result, we think that this article adds to the related social psychology and sociology literature (Bonanno et al., 2007; Hoang & Knabe, 2021; Navarro-Carrillo et al., 2020; Tan et al., 2020; Winkelmann, 2009), to which we provide a robust piece of evidence. We contribute to a recent strand of literature on economics on how experiencing major events may shape preferences and beliefs over a variety of outcomes, from risk aversion to political preferences (Laudenbach et al., 2019; Malmendier & Nagel, 2011; Malmendier, 2021a, 2021b). Depression is characterized by self-debasing ideas and a loss of interest in pleasurable activities (Beck & Alford, 2009) that can be considered a change in beliefs and preferences (de Quidt & Haushofer, 2019).

This paper also adds to the literature on mediators and moderators of the effect of income losses on stress, anxiety, and depression. The role of cognitive factors in mediating the onset of mental health is an understudied area (Beck & Alford, 2009): by using a set of validated measures of expectations, preferences, and fears, we provide a piece of evidence. Physical health and comorbidity have been further investigated. The literature has received valid contributions during the current pandemic (Belot et al., 2021; Etheridge & Spantig, 2020). Our results do not replicate the main findings in the literature, but it is possible that in the context of negative economic shocks, the mechanisms may be different. We further contribute to the burgeoning literature on Covid19. The most relevant references are summarized in the next section. The current study attempts to fill a gap by trying to provide causal evidence.

This paper proceeds as follows. Section 2 illustrates the contribution to the existing literature. Section 3 presents data and empirical strategy. Section 4 discusses Results. Concluding remarks are in Sect. 5. An appendix includes additional statistical analysis. The complete questionnaire of the study is available at the OSF page of the companion working paper (https://doi.org/10.31235/osf.io/wx9d4).

## Review of the state of the literature

For the purpose of this article, a labor market shock is a sizable income reduction originated by an event occurred in the labor market, such as losing the job or a significant source of earnings, being injured or getting sick. A labor market shock belongs to the category of major negative events that may shape behaviour (Bauer et al., 2016; Bogliacino & Montealegre, 2020; Bogliacino et al., 2017; Haushofer & Fehr, 2014; Malmendier & Nagel, 2011; Mani et al., 2013). Physically experiencing similar events differs from receiving information about them: negative events may have long-term consequences on behaviour due to the suggested mechanisms of scarring effects or synaptic tagging (Kozlowski et al., 2020; Laudenbach, et al., 2019; Malmendier & Sheng Shen, 2019). Stress, anxiety, and depression document the pathological consequences of suffering negative shocks and a profound reassessment of beliefs and preferences. Similar results hold for school and mass shootings (Lowe & Galea, 2017; Rossin-Slater, et al., 2020).

Sociology and social psychology have long analysed how structural and economic conditions impact individuals’ subjective experiences and provide insight into possible causal paths.

More in details, in sociology, Durkheim (1951) analysed anomic suicide as resulting from generalised conditions of social and economic crisis, a thesis further developed by Merton (1938)’s study of the experience of social structure as feelings of anomie. In social psychology, Jahoda, after a first seminal sociographic description of the individual experience of unemployment (Jahoda et al., 1971), developed the Latent Deprivation Model (Jahoda, 1981, 1982) of unemployment, according to which unemployment deprives individuals not only of income but also of latent and intangible benefits such as social status, prestige, structured time use, a sense of collective purpose, social contact, and activity. This model was tested in a large body of empirical studies distinguishing between the pecuniary from the non-pecuniary costs of unemployment (Young, 2012). Indirect evidence on the role of labour market and social status comes from studies on resilience, where SES is systematically associated with poor physical and mental health outcomes (Bonanno et al., 2007). Recent meta analyses and literature reviews suggest the evidence is well established (Cutler et al., 2012; Navarro-Carrillo et al., 2020; Tan et al., 2020).

Still in the social-psychological tradition, Seligman (1975) posits that pessimistic beliefs on self-efficacy may lead to vicious cycles, in which helplessness leads to depression. Recent evidence of the same mechanism has been provided for Australia (Cole et al., 2009) and Germany (Clark et al., 2001). Employed and unemployed have different experience of psychological and emotional well-being (Clark, 2003) while performing the same activities (Hoang & Knabe, 2021), which translate into different outcomes with respect to mental health (Flatau et al., 2000). Moreover, the relationship between unemployment and well-being is robust to the inclusion of social capital (Winkelmann, 2009).

As summarised in two reviews (Milner et al., 2013; Wanberg, 2011), in psychology, there is a vast body of empirical literature on the individual consequences of unemployment and income shocks focusing on their effects on mental and physical health, suicide, and well-being. The empirical literature converges in showing that sudden loss of jobs and income has marked negative consequences on the well-being of unemployed individuals (see for instance, the meta-analytic study by McKee-Ryan et al., 2005 and the cross-country study by Cygan-Rehm et al., 2017). As per the mechanism through which this occurs, the main mediator is financial strain that triggers loss of control, lowered self-esteem, social withdrawal, and family disruption that undermine mental health (Brand, 2015). Using a two-year longitudinal study of 756 subjects, for instance, Price et al. (2002) have shown that Financial Strain (FS) and Personal Control (PC) are the two fundamental mechanisms linking job loss to adverse mental health and physical health outcomes. FS mediates between job loss and depression, while reduction of PC moderates the impact of FS and depression on poor functioning and health.

A relatively large epidemiological literature informed this discussion on the evolution of health during major adverse shocks, such as recessions and depressions. This interest was revived in the last decade by the Great Recession (2007–2009), the Euro crisis (2010–2013) and the austerity measures adopted in Europe (Greece in particular) as a result (Barr et al., 2015; Economou et al., 2013; Gili et al., 2012; McInerney et al., 2013; Thern, et al., 2017; Wang & Fattore, 2020). Previous contributions have examined business cycles and mortality rates, including suicide as a proxy for psychological wellbeing (Ogburn & Thomas, 1922; Ruhm, 2000). Aggregate patterns of association between unemployment or inequality and mental disease or suicide may be criticized, as they fail to control for several area-specific unobservables (Wang & Fattore, 2020). A similar argument can be raised over the studies that look at prevalence of mental disorders along socio economic status (Bromet et al., 2011; Lund et al., 2010, 2011).

The findings that white middle-aged Americans have been facing ‘death of despair’ (i.e., alcohol-induced liver pathologies, suicide, and opioids overdose), have raised further discussion as to whether or not the Great Recession played a significant role (Case & Deaton, 2015, 2020), though the authors do believe it did not.

Furthermore, since we completed our longitudinal survey, the literature on Covid-19 and mental health has burgeoned and there are already several meta-reviews and meta-analyses (Loades et al., 2020; Nochaiwong et al., 2021; Serafini et al., 2020; Vai et al., 2021). However, from such reviews and from selectively reviewing some contributions, we notice that most studies are simply observational and aim to measure the prevalence of mental health problems during 2020 and 2021. In the UK, for instance, three studies based on large samples (Hampshire et al., 2021; Jia et al., 2020; Niedzwiedz et al., 2021) compare the before and after situations to provide a picture of the increase of mental health problems prevalence as result of the Covid-19 and related NPIs. For instance, Niedzwiedz et al. (2021) show that psychological distress increased one month into lockdown, rising from 19.4% in 2017–2019 to 30.6%. Hampshire et al. (2021) based on a sample including 379,875 people in the United Kingdom show an increase in the prevalence of mental health problems, with a more marked increase for older adults and people with lower incomes. A survey conducted in the US shows that the prevalence of depression symptoms was more than threefold higher during Covid-19 than before (Ettman et al., 2020). A survey in Italy found that Depression and anxiety symptom prevalence was 24.7% and 23.2%; 42.2% had sleep disturbances, and 17.4% reported moderate/severe insomnia (Gualano et al., 2020). In most of these studies, the outcome is self-reported using standardized scales.

While these studies corroborate our findings concerning the prevalence of mental health problems, to the best of our knowledge, they do not focus on the concept of shock as defined in this paper, do not look at the specifics of labor market shocks and, especially, they do not attempt at retrieving causal effects using explicit identification strategy. One exception is a study of the effects of curfew in Turkey that adopted a regression discontinuity design (Altindag et al., 2022). This study found that the decreased mobility induced by the curfew caused psychological distress especially because of increased social and physical isolation. Yet, this applies to a different age group: as the curfew was applied only to the cohort 65 and older, to a large extent it applied to pensioners. Instead, we argue that this study attempts to fill a gap in the literature and can be clearly differentiated from existing Covid-19 literature on mental health. Another exception is a study on the US on the impact of stay-at-home policies, which leverage on the differences across states to document a worsening of mental entirely driven by women (Adams-Prassl et al., 2022). Finally, a study in Pakistan finds that a job loss during Covid-19 is associated with significant increase in adult mental distress and children depression symptoms (Baranov et al., 2022).

## Data and analysis

### The database

Data for this paper come from the longitudinal study by Bogliacino et al. (2020). When Italy issued the first mandate to lockdown the economy (followed by Spain), the research team explored the possibility to contribute to the public debate analysing different aspects of the undergoing crisis.

The data collection aimed at interviewing three times a sample of 1000 participants in each of three countries. Italy, UK, and Spain were chosen partly because most severely hit by the pandemic in the first phase, and partly because, allegedly, they followed different strategies, or at least initially put forth different strategies. The three data points would have been collected at intervals of 8–10 days, guaranteeing a longitudinal dimension. The research plan envisaged specific modules each week and a set of repeated questions. During the period of the study, all three countries were under severe mitigation strategies, which were slowly released at the end of the data collection period. Data collection covered April–May 2020.

In week one, participants took part to a list experiment to identify the support for different containment strategies. The list experiment was carried out to control for Social Desirability Bias in a period in which the framing “Health versus the economy” made it politically incorrect to express doubts on the costs of the Covid-zero policies (Codagnone et al., 2020). Week one also included a profiling involving both health status and socio-economic conditions. The second week focused on an experimental manipulation to detect the impact of shocks on cognitive function and preferences (Bogliacino et al., 2021). The third week questionnaire evaluated which style of institutional communication was more effective to convince citizens to support a specific containment policy under conditions of uncertainty, value conflicts, and distributional conflict, to improve communication strategies and behavioural management, and included a set of measurements on behavioural change (Codagnone et al., 2021).

The sample is randomly drawn from an online panel representative of the population in three countries: Italy, Spain, and the United Kingdom (UK). The representativeness of the panel is based on matching the population on a limited number of observables. The interviews occurred first in the week from 24 April to 1 May (Wave I, N = 10,551: 3,504 in Italy; 3,524 in Spain; 3523 in UK), in the week from 2 to 9 May (Wave II, N = 4,890: 1,652 in Italy; 1,660 in Spain; 1578 in UK), and finally from 10 to 20 May (N = 3191). For the final sample, we collected responses from 3,191 participants: 1,032 in Italy, 1,021 in Spain and 1,138 in the UK. During the period of study, all three countries were under severe mitigation strategies, which were slowly released at the end of the data collection period.

Four questions are shared across the waves. The first longitudinal question is related to the difficulties of fully isolating during the lockdown (“How much would the following factors prevent you from fully isolating yourself?”), including five items (1. need to earn an income; 2. need to take care of others outside the home; 3. do not want to miss certain social events; 4. urge to practice sports; 5. urge to leave the house), with answers collected on a four-point Likert scale, from Very Unlikely to Very Likely. From this first question, we assign a score to each item from one to four and calculate the average of the five components to build an index. The second question (“Over the past week, have any of the following events happened to you?”) investigates the negative events that occurred during the previous week, including nine items (1. forced to stay at home or in a shelter; 2. employment loss; 3. a decrease in earnings; 4. need to home-school; 5. unable to access healthcare; 6. unable to access sufficient food; 7. tested positive for Covid-19; 8. applied for governmental support; 9. sought help from charities), with Yes or No responses collected. We build an index summing the number of positive responses to this question. The third question is our main outcome variable (“How often have you been bothered by the following over the past seven days?”) and measure stress, anxiety, and depression. We adapted the DASS-21, Depression, Anxiety and Stress Scale – 21 Items (Lovibond & Lovibond, 1995) and the Stanford Acute Stress Reaction Questionnaire, SASRQ (Cardeña et al., 2000). A similar version of this scale is in Hensel et al., (2022). We use an eight-item scale, with answers collected on a Likert scale from one to four, and where each point corresponds to a specific number of days during which the symptoms have been experienced over the previous week. We rescale each item from low to high intensity of exposure, and we average the response across the eight items. The last longitudinal question asks for change in the labour market conditions and is a standard validated question (“How has your wage/earnings been affected after the COVID-19 outbreak?”). We built a dummy equal to zero if there was no change and one if a loss was experienced, i.e. this is an indicator variable for reporting a reduced pay, a termination of the contract, a temporary or definitive closure of the business, or a temporary interruption of the payment by the company.^2^

These are the other questions that have been used in the paper. Participants in the first week answered a set of baseline socio-demographic and health status questions, derived from standard scales. We used a question to measure financial strain (“how long they can pay their bills if they do not receive any income?”) and their self-reported health status (“How is your health in general?”). In the second week, we asked participants, as a special module, a subset of the Global Preferences Survey by Falk et al. (2018): this is a set of experimentally validated questions to measure risk and time preferences, altruism, trust, positive and negative reciprocity.^3^ From the questionnaire in the third week, we used two sets of questions: the first set measured expectations using three questions; two on optimism over the economic outlook and personal job outlook (taken from Eurobarometer, “What are your expectations for the year to come: will 2021 be better, worse or the same, when it comes to the economic situation (in our country)/your personal job situation?”) and one on the length of the recovery (standard formulation, “What are your expectations on the recovery from the current pandemic: when do you think we will go back to our normal lives like before the outbreak?”). The second block measured the level of fear (“Among the following negative scenarios for the future, please indicate how likely you consider them?”) of three future scenarios (1. a new outbreak of the disease; 2. an economic depression; 3. a permanent restriction to individuals’ rights and freedom), and participants answered on a Likert scale from one to four.

The OSF page by one of the authors provides all the relevant information regarding this data collection (Bogliacino et al., 2020). The Institutional Review Board of the Universitat Oberta de Catalunya approved the protocol. All participants provided informed consent.

### Econometric strategy

We estimate the following model:

1 $${y}_{it}=\alpha +\beta {D}_{i}\mathbf{I}\left(t=\mathrm{2,3}\right)+\rho {T}_{i}^{1}\mathbf{I}(t=\mathrm{2,3})+{\gamma }_{t}+{\delta }_{i}+{\varepsilon }_{it}$$

where *i* indicates the participant, *t* the wave (t = 1, 2, 3), *y* is the outcome variable, ${D}_{i}$ is the variable equal to one if the participant *i* experienced the shock at wave II or III, $\mathbf{I}\left(t=\mathrm{2,3}\right)$ is an indicator function equal to one for the second and third wave, ${T}_{i}^{1}$ is a dummy equal to one if the participant experienced the shock in the first wave, ${\gamma }_{t}, {\delta }_{i}, {\varepsilon }_{it}$ are, respectively, wave fixed effects, participants fixed effects and time-variant unobservables.

To identify the effect of the labour shock, we leverage the variation experienced by participants who did not suffer a shock in wave I but reported it in wave II or III. Of the 3191 respondents, 1276 were never treated, 286 were treated in wave II or III, and 1629 had already been treated in wave I.

Our data include four cohorts of units: the never treated (*C*_*0*_), those reporting a shock in the first data collection (*T*_*1*_), those reporting a shock in the second data collection (*T*_*2*_), and those reporting a shock in the third data collection (*T*_*3*_). We cannot separate the invariant characteristics from the shock for T_1_, so we allow for a group-specific trend. Nonlinearity of their trend would not affect our results as we are never using them as a comparison.

Identification relies on the assumption of conditional parallel trends; that is, there is no time-varying outcome shifter correlated with the assignment to shock at wave II or III. If this assumption holds, we can subtract the counterfactual trend, estimated from those who were not treated, and recover the causal impact of the treatment *on those who received the shock.*

**Fig. 1:**
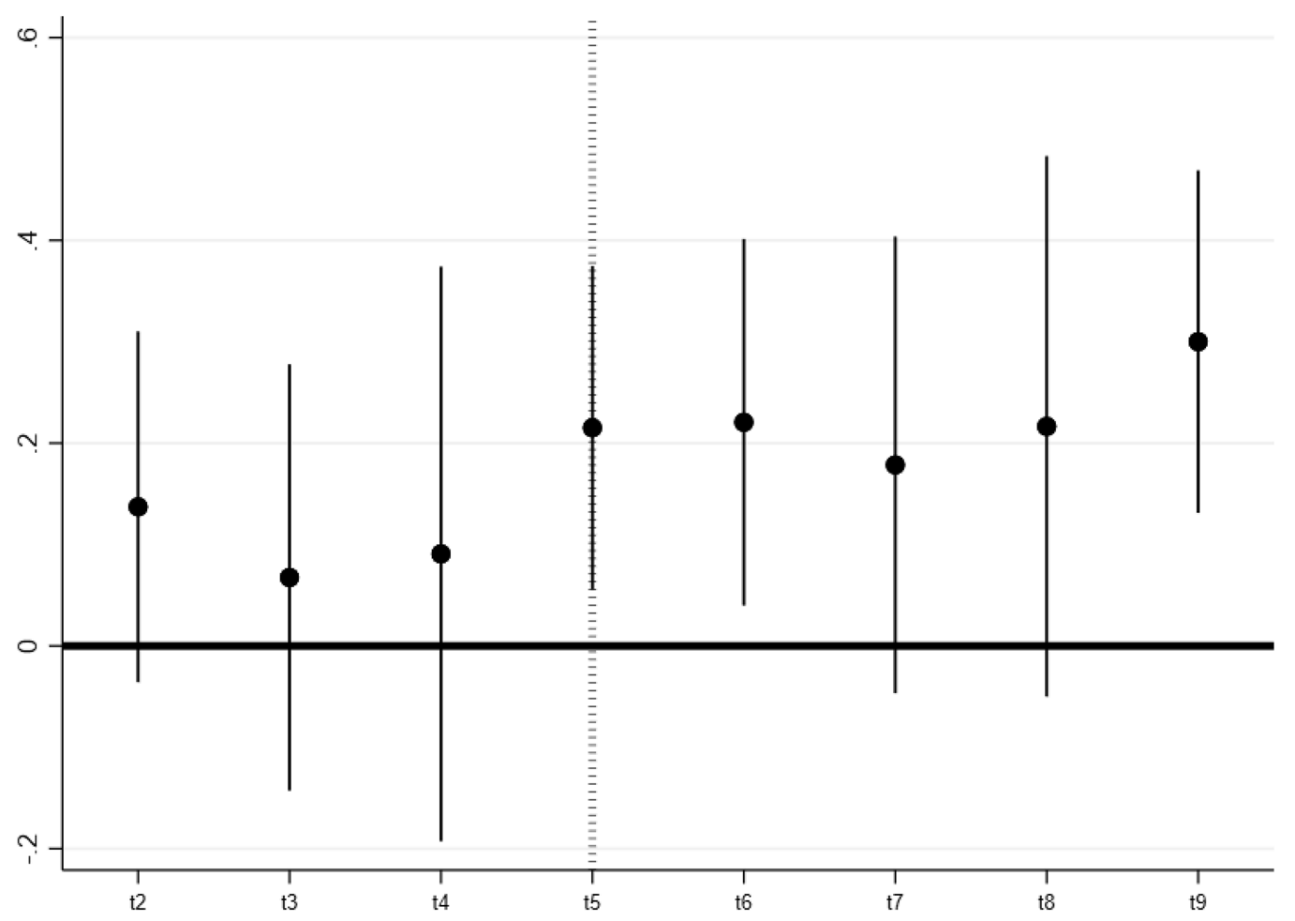
Assessing Pre trends. Data from the Longitudinal Study by Bogliacino et al. (2020). t1 is the first two days of data collection, t2 the two following days, etc. Each dot is a coefficient of the interaction between the date dummy and the treatment dummy (equal to one for those that received a labour shock at waves II or III). t5 is the date of the shock. Fixed Effects Estimation. Confidence interval at 95%

The first step to assess the plausibility of identification is to discuss pre-trends. Although the absence of pre trends does not imply parallel trends, it certainly weighs in favor of its plausibility. We can exploit a feature of our dataset, the fact that we have the exact day in which each person answers the interviews. Using this information, we can see whether the *T*_*2*_ and *T*_*3*_ groups were on average statistically different from the never treated (*C*_*0*_) in the days before the arrival of the shock. We can plot the coefficients of the interactions between the calendar day of the interview and the dummy for treatment and see whether they are statistically different from zero, to eventually conclude that the treated were not diverging from the control before receiving the treatment. This evidence is presented in Fig. 1 below.

The event study just described has a pro and a con. The second week coincides with the end of the month. If workers expect termination, they will anticipate the effect, violating identification. Simply put, they will become more stressed already before being hit by the shock. Since the occurrence of firing is more probable at the end of the month, testing for differences in the first week of the study becomes a powerful assessment of anticipation effects*.* The con is the following: different participants are interviews are different calendar days, which means that when looking at the coefficients of these interactions, the pool of people compared with the control changes. Overall, we think that this is a minor concern because it plays in favor of finding a difference, so it can become a strength if the coefficients turn out to be non-significant.

Besides anticipation and pre trend, the main threat comes from time-varying variables contemporaneous to the shock, which may confound the inference. Having staggered treatment makes the design more credible. However, the popular Two-Way Fixed Effect estimator (TWFE) may carry negative consequences in this setting. In practice, FE estimates are a weighted average of many 2 × 2 difference in differences, where treated individuals are compared with groups of participants whose treatment timing does not coincide. Some of these controls are in reality, already treated units (Baker et al., 2022). Even if the parallel trend assumption holds, if the effect is not one-shot or is heterogeneous at a different point in time, TWFE provides a biased estimate. The way we respond to this problem is the following. We separately use *C*_*0*_ and *T*_*2*_ and *C*_*0*_ and *T*_*3*_ and estimate three causal parameters: the effect of treatment on *T*_*2*_ between wave I and wave II, the effect of treatment on *T*_*2*_ between wave I and wave III, the effect of treatment on *T*_*3*_ between wave II and wave III. This is similar to what is done in the literature on minimum wage where the different policy innovations are studied separately (Cengiz et al., 2019).

Another threat comes from Covid-19 policies. The policies to contrast the aggregate shock may be heterogeneous. In one of our specifications, we introduce day-by-country fixed effects. Since policies vary at the country-by-day level, this fixed effect should absorb this variation.

Finally, we analyse the role of measurement error. If we failed to detect the timing of the occurrence of a shock, there would be a classification errors across groups. For the sake of the argument, assume that some participants classified as *T*_*2*_ where actually shocked at time one (*T*_*1*_) but misreported the correct timing of the event. In our research design we used the variation of those hit at time two or three with respect to those who did not receive shocks, whereas the *T*_*1*_ respondents are allowed to have their own time trend. Mixing *T*_*1*_ and *T*_*2*_ may induce an inferential error. We can try to address this problem by using an alternative measure of shock. Although the responses to the two questions by the same participants are clearly not independent, two comprehension errors are at least very unlikely, since the phrasing is different.

We exploit another feature of our data: we posed another question over the occurrence of adverse events (see the discussion in the data section above). The latter are all associated with negative productivity consequences (lack of mobility, health issue, etc.) and thus impact the labour market. We can apply the same research design, using this variable to identify the treatment. The methodological choice is how to define a shock: given that many adverse events can occur, we should be able to estimate how these adverse events map into an income loss. We use two definitions: according to the conservative definition, a treated individual suffered more than a standard deviation of negative events with respect to the first week; according to the lax definition, a treated individual is that who suffered one additional event with respect to the first week.

### Attrition and relationship with other papers from the same project

Before presenting the main results, two aspects are worth discussing. The first concerns the role of attrition in data collection and its effect in terms of external and internal validity. The second is this paper’s role within the overall project and the relationship with the other articles written on the same data.

Attrition occurs when respondents drop out of the data collection. In a longitudinal study, we fail to observe and measure outcomes for the portion of the population that drops out. We first discuss the potential harm to external validity. Attrition provides a threat. For example, in Panel B of Appendix Table 6 in the Appendix, we report the sample quotas according to three dimensions for the initial random sample: sex, age, and region of residence. In Panel A of the same Table, we report the corresponding shares for the population aged 18–75, taken from Eurostat. In terms of these observable characteristics, the sample is representative of the population of interest. Nevertheless, attrition from wave I (where representativeness holds) is not random (it is the result of a decision made by subjects); thus, projecting to the overall population our outcome is unlikely to provide an unbiased estimate of the true average effect.

We now discuss internal validity. Within an RCT, selective attrition out of treatment and control represents a confound of the intervention. Stated simply, attrition and intervention change at the same time and we cannot assign the effect to either of the two. The research design of this study is not an RCT, though: in fact, we never claimed that in absence to treatment, the outcome for T_2_–T_3_ with respect to C_0_ would be the same (*mean independence*). The DiD design assumes that conditional on the fixed effect, the assignment is as good as random: in other words, the evolution of the outcome in the treated groups would have been the same as in the control, except for the shock (*parallel trends*). The relevant causal parameter of interest is the ATOT, not the ATE.

In this setting, including data from those who dropped out makes things worse. It would introduce a time-varying change in composition between the treated and untreated, harming identification (Hong, 2011). Matching untreated with treated on observables would also make things worse. Matching on observables would require time-invariant unobservables to be balanced, but this is more stringent than our identifying assumption.

Having established that the identifying assumption is parallel trends, and not mean independence, the question on attrition can be rephrased as follows: Is parallel trend more likely to hold in our sample, or in a hypothetical scenario where the initial sample would have stayed until the end? In the latter scenario, we could of course count on a larger number of T_2_–T_3_ and C_0_. But more data does not mean better data. The identifying assumptions are untestable but should be plausible (Heckman, 2010). Some indirect evidence over plausibility would come from checking pretrends on dropout and look at how close they are with the two groups (T_2_–T_3_ and C_0_), but we lack the information for this assessment.

For the sake of completeness, we report the differences between dropouts, T_2_–T_3_ and C_0_ in terms of demographics and comorbidity. In the Appendix, we include in Appendix Table 7 a multinomial logit regression of sociodemographic and health status over the likelihood to be classified as T_2_–T_3_ and C_0_ with respect to having dropped out. The results are mostly unsurprising: T_2_–T_3_ and C_0_ are different from dropouts in terms of the same characteristics and with very similar coefficients: age, income, labor market status, residential neighbourhood, having kids at school. The only coefficients that are statistically different across equations concern home ownership, which is different between T_2_–T_3_ and dropouts but not between the latter and C_0_, (${\chi }^{2}$=2.18, p = 0.06) and household size (the other way around, ${\chi }^{2}$=2.18, p = 0.06). None of the two are significant at 5% and would not survive a control for multiple hypotheses testing (which here is clearly an issue).

Summing up, estimating the model on those for which we have three observations is the correct procedure but it is not a guarantee of parallel trends, and additionally, it affects the extent to which the results can be generalized to the overall population. How do we deal with this last problem? Since this is an average treatment on the treated, it requires further assumptions on the attrition and the original assignment of the shock to be generalised to the overall population. We could infer some conclusions on the average treatment effect under more stringent assumptions. Assume heterogeneity of the treatment effect between cells of the population. The average treatment effect becomes the weighted average of the treatment effect within each cell. To extrapolate, we need the probability of being treated to be positive in the original sample (*common support*). Additionally, we must assume that attrition is independent of the shock, conditional on all observable variables gathered in the first wave. Conditional on those observable variables collected in the first wave, we can compute the likelihood of survival until wave III. Analytical weights are the inverse of this estimated survival probability. Codagnone et al. (2021) performed that exercise using a random forest model. We will perform this weighted regression in the Results section.

As mentioned in the introduction, the analysis for this article uses data collected between April and May 2020, in the context of a longitudinal study in three waves (Bogliacino et al., 2020), surveying respondents in three countries (Italy, Spain, and the UK). The last part of this section describes the project and the other articles from it to clarify the contribution from the current article better.

The project addressed four topics: (1) the trade-off between economy and health in the management of the pandemic (Codagnone et al., 2020); (2) the consequences of exposure to shocks on cognitive performance, preferences, and behavioural change (Bogliacino et al., 2021; Codagnone et al., 2021); (3) the role of experts’ endorsement in obtaining approval for policy interventions (Bogliacino et al., 2021); (4) the risks of mental health (Codagnone et al., 2020).

The only contribution whose research question presents some overlap is Codagnone et al. (2020). The latter tried to predict the likelihood of being under severe stress, anxiety, and depression based on socio-economic characteristics and vulnerabilities. Codagnone et al. (2020) only used data from the first wave and projected the likelihood of being under severe stress using a random forest algorithm but made no claim on identifying the causal effect of shocks. Instead, Bogliacino et al. (2021) and Codagnone et al. (2021) claim causality. However, the former looked at how shock-driven stress and negative emotions affect cognitive performance and preferences, while the latter at how expectations, fear, and behavioral change (risky behavior, social capital, and cultural consumption) responded to shocks. Bogliacino et al. (2021) examined an entirely different research question and used a standard randomised research design.

## Results

Table 1 presents the estimation of Eq. (1). Columns (1) to (3) present OLS estimates with clustered standard errors (at the respondent level). The coefficient of interest is the interaction of the treatment dummy with a dummy equal to one if the observation is posterior to the shock. In columns (1) and (2), we include wave fixed effect, whereas in column (3) we add calendar day fixed effect (technically a dummy per each two consecutive days of data collection). Column (2) differs because we allow the interaction effect to varying between the first and the second period after the shock. Since we have participants who suffered shocks in the first wave, we introduce a dummy equal to one for those belonging to this group and allow for their specific time trend.

**Table 1:** The effect of negative labour market shock on mental health

	(1)	(2)	(3)	(4)	(5)	(6)	(7)
Outcome: stress anxiety and depression	OLS	OLS	OLS	FE	FE	FE	FE
Labor shock X I[t = 2]		0.13** (0.06)			0.11*** (0.04)		
Labor shock X I[t = 3]		0.13*** (0.04)			0.12*** (0.04)		
Labor shock X I[t = 2,3]	0.12*** (0.03)		0.12*** (0.03)	0.12*** (0.03)		0.18*** (0.03)	0.18*** (0.03)
Constant	1.70*** (0.02)	1.70*** (0.02)	1.68*** (0.03)	1.90*** (0.01)	1.90*** (0.01)	1.90*** (0.02)	1.39*** (0.07)
Observations	9573	9573	9573	9573	9573	9573	9573
Number of iid				3191	3191	3191	3191
R-squared	0.04	0.05	0.05	0.01	0.01		
Labor shock in wave 1	Yes	Yes	Yes				
Labor shock in wave 2–3	Yes	Yes	Yes				
Wave fixed effects	Yes	Yes		Yes	Yes		
Fixed effects				Yes	Yes	Yes	Yes
Day by country fixed effects							Yes
Calendar fixed effects			Yes			Yes	

Data from the Longitudinal Study by Bogliacino et al. (2020a). Outcome: Index of mental health. Labour shock is equal to one if the shock occurs after wave I. I[t = k] is a dummy equal to one for observations from wave k.

*OLS* Ordinary Least Squares, *FE* Fixed Effect.

Clustered standard errors in parentheses ***p < 0.01, **p < 0.05, *p < 0.1

TWFE estimations are reported in columns (4) to (7). Column (4) controls for wave fixed effects. Column (5) allows the effect of the shock to differ between the first and second period after the treatment, column (6) controls for calendar day fixed effect, and column (7) for day-by-country fixed effect.

The results are very stable and robust. In column (1), the shock increases the levels of stress, anxiety, and depression by 0.12 points of our index. The index runs from zero to one, and we treat it as continuous. To quantify the effect, we compute the standard deviation in waves II and III of those who did not receive a shock (0.75). If we standardise the effect, it corresponds to 16% of a standard deviation. The outcome averages out for how long the participant experienced a set of symptoms during the previous week. The Likert scale includes four items: most or all the time (5–7 days); occasionally or a moderate amount of time (3–4 days); some or a small amount of time (1–2 days); rarely or none of the time (less than 1 day). Each scale point corresponds to experiencing the symptoms for approximately 1.5 days. The effect amounts to four additional hours. Notice from column (2) that the effect is stable in the first and second weeks after the shock. Controlling for time effects at a higher level of granularity does not affect the magnitude of the effect (column (3)).

TWFE further controls for all time-invariant unobservable characteristics of the participants. The magnitude of the effect becomes larger, reaching 24% of a standard deviation, using the same normalization as before. This result is robust even when we include day-by-country fixed effects, which can control for the change in Covid-19 policies (column (7)).

In the Appendix Table 8, we further allow for time varying effects of gender, education and income rank. The estimated impact is completely unaffected even when we control for time and day-by-country fixed effects.

As we explained in Sect. 3.2, we assess pre trends with an analysis in the spirit of an event study. We interact the treatment status with calendar day dummies before and after the treatment, estimated via TWFE. The calendar days refers here to the day in which the interview was performed (different people were interview at different time during the same week). Figure 1 plots the coefficients for those interactions. These effects are relative to the first two days of data collection, and the graph covers the week before and after the shock in wave 2. We cannot reject the lack of pre-trend. As we mentioned in the previous section, the beginning of the second week coincides with the end of the month and thus would be the best candidate to observe anticipation effects. Data reject their presence.

Albeit the presence of different timings of treatment makes identification more plausible, as other shocks confounding the effects should coincide with the labour market ones at more than one time, it also introduces potential heterogeneity. TWFE uses information from previously treated units to estimate the counterfactual trend. If the causal effect occurs in the form of a local jump of the outcome variable or is homogeneous among groups treated at different times, the estimator would still be unbiased. If, instead, there is heterogeneity or dynamic treatment effect, the estimator becomes biased because the counterfactual trend would be estimated also with the post treatment evolution of the outcome. The solution proposed by Callaway and Sant’Anna (2021) consists in relying on never treated (C_0_) as a control group and estimating group average treatment effects (T_2_ versus C_0_ and T_3_ versus C_0_) as if we were running a DiD for each cohort of treated individuals. We follow the notation in their original paper and use GATT [g, t] to indicate the change in outcome between wave g-1 and t for the group treated in wave g. Callaway and Sant’Anna (2021) suggest various ways to consolidate the different effects using weights estimated with a propensity score. However, given our limited groups and time structure, we provide separate estimates of the effect for T_2_ and T_3_. Results are reported in Table 2. The effect for those treated in wave II is stable at 16% of a standard deviation, while it reduces to 10% for those treated in wave III. Also, this last coefficient is not statistically significant due to the sample size.

**Table 2:** The effect of negative labour market shock on mental health during the pandemic. Group Average Treatment Effects

	(1)	(2)	(3)
	FE	FE	FE
GATT [2, 2]	0.12*** (0.04)		
GATT [2, 3]		0.13*** (0.04)	
GATT [3, 3]			0.08 (0.08)
Constant	1.71*** (0.04)	1.70*** (0.03)	1.62*** (0.02)
Observations	2938	2938	2738
Number of iid	1469	1469	1369
R-squared	0.03	0.02	0.01
Calendar fixed effects	Yes	Yes	Yes
Individual fixed effects	Yes	Yes	Yes

Data from the Longitudinal Study by Bogliacino et al. (2020). Outcome: Index of mental health. Labour shock is equal to one if the shock occurs after wave I. I[t = k] is a dummy equal to one for observations from wave k. GATT[g,t] is the Group Average Treatment effect for those treated in wave g between time g-1 and t

*FE* Fixed Effect

Clustered standard errors in parentheses ***p < 0.01, **p < 0.05, *p < 0.1

After establishing the main result, we analysed the role of measurement error. Misreporting of shocks would bias the estimate. Misreporting is costless given the non-administrative source of our data. However, we gathered information on a set of adverse events (get tested, seek help, home-schooling, apply for subsidy), which produce direct or indirect consequences on productivity and may translate into a negative labour market shock. We performed two estimations: in one, we defined as treated those that received at least one additional adverse event with respect to the first week. In the second, we label as treated those participants that received at least two adverse events with respect to the first week (the standard deviation of this variable is 1.8).

Table 3 shows the difference-in-differences model, with and without fixed effect, using the two measures based on the exposure to negative events. The results are extremely close to Table 1, with the magnitude of the effect ranging from 14 to 20% of a standard deviation.

**Table 3:** The effect of negative labour market shock on mental health during the pandemic

	(1)	(2)	(3)	(4)
Outcome: stress anxiety and depression	OLS	OLS	FE	FE
Negative events [+1] X I[wave 2–3]	0.11***		0.11***	
	(0.02)		(0.02)	
Negative events [+1sd] X I[wave 2–3]		0.16***		0.16***
		(0.03)		(0.03)
Constant	1.86***	1.87***	1.90***	1.90***
	(0.02)	(0.01)	(0.01)	(0.01)
Observations	9573	9573	9573	9573
Number of iid			3191	3191
R-squared	0.01	0.02	0.02	0.02
Negative events [+1]	Yes			
Wave fixed effects	Yes	Yes	Yes	Yes
Negative events [+1sd]		Yes		
Individual fixed effects			Yes	Yes

Using negative events as a measure of shocks

Data from the Longitudinal Study by Bogliacino et al. (2020). Outcome: Index of mental health. Labour shock is equal to one if the shock occurs after wave I. Negative events [+1] is a dummy equal to one if a participant reported at least one more negative event in waves II and III with respect to wave I. Negative events [+1sd] is a dummy equal to one if a participant reported at least one point eight more negative event in waves II and III with respect to wave I. I[t = k] is a dummy equal to one for observations from wave k. Clustered standard errors in parentheses

*OLS* Ordinary Least Squares, *FE* Fixed Effect

***p < 0.01, **p < 0.05, *p < 0.1

We now explore heterogeneity along the following dimensions: financial strain, health status, preferences-beliefs-fears, and difficulty to isolate. The rationale for this analysis is that learning about differences in causal impact can inform policies to prevent or soften those impacts.

We used two research designs for this part. The first is limited to the data on the difficulty to isolate, where we collected the longitudinal variation across the waves. As a result, we can run the same analysis as Eq. (1), but replacing the previous dependent variable with the difficulty to isolate. For the other variables used for the heterogeneity analysis (financial strains, health status, preferences-beliefs-fears), we have only one measurement, thus we use a triple difference (Gruber, 1994) design. For a given mediator X, computed as a dummy variable, we ask whether the causal effect of shocks on mental health is different when the participant scores high (versus low) on X. All estimations include three double differences:

- the interaction between the treatment and the dummy for waves II and III;
- the interaction between the treatment and the mediator;
- the interaction between the mediator and the dummy for waves II and III.

We do not interpret the effect as causal, because potential confounds may exist and because the requirement of power increases significantly.

We now detail how we measure each mediator.

Physical health is a dummy from a self-reported health status, coded as one if the status is not reported as good or very good. Financial strain is computed from the question on the difficulty to pay bills and is equal to one for participants whose saving and financial net would cover the financial obligations for less than three months.

Using the set of questions from wave III, we can compute a measure of worries for the future. We use the three questions related to the perceived likelihood of a future outbreak, an economic depression, a permanent restriction on rights and freedom and the question of pessimism about the future. We transform the two questions on pessimism into dummies, equal to one for those seeing a worse prospect. The rest of the variables maintain the original coding. To build a standardized index, we use the predicted score for the first component from principal component analysis. The associated eigenvalue is 2.2, explaining 36% of the total variability, and the Kaiser–Meyer–Olkin measure of sampling adequacy is 0.68 (acceptable). The predicted score is centred in zero. We split the sample above and below zero.

From the preference module in wave II, we compute a dummy to capture those participants that are more risk oriented, future oriented and pro-social. More self-control, less risk aversion, and more pro-sociality constitute a more resilient mindset (Connor & Zhang, 2006; Luthar & Zelazo, 2012). We extract the first component from the set of responses (associated eigenvalue 2.02, explained variance 28%, KMO measure of sampling adequacy 0.70). The predicted score is centred in zero. We split the sample above and below zero.

Table 4 presents both OLS and FE estimation. We can never reject the null hypothesis. Since the inclusion of multiple interaction terms induces collinearity, we perform equivalence tests (Lakens, 2017) to assess whether the lack of significant effect is non-equivalent to zero. We use the two one-sided t-tests to assess whether $\left|t\right|\ge \varepsilon$, where *t* is the corresponding t-statistics computed for the coefficient of interest and $\varepsilon$ is a properly defined threshold (in this case, we use 2, as this would mean an effect within 70% of a standard deviation). The procedure consists of testing whether the effect is larger than the pre-defined threshold. The test uses the OLS model.

**Table 4:** The effect of negative labour market shock on mental health during the pandemic: Mediators and moderators

	(1)	(2)	(3)	(4)	(5)	(6)	(7)	(8)	(9)	(10)
	OLS	FE	OLS	FE	OLS	FE	OLS	FE	OLS	FE
Outcome variable	Stress anxiety and depression								Difficulty to isolate	
Labour Shock X Fin Strain X I[t = 2,3]	0.03 (0.07)	0.03 (0.07)								
Labour Shock X Bad Health X I[t = 2,3]			− 0.08 (0.07)	− 0.08 (0.07)						
Labour Shock X Worried X I[t = 2,3]					− 0.05 (0.07)	− 0.05 (0.07)				
Labour Shock X Mindset X I[t = 2,3]							− 0.03 (0.07)	− 0.03 (0.07)		
Labour Shock X I[t = 2,3]									0.06 (0.04)	0.06 (0.04)
Constant	1.60*** (0.02)	1.90*** (0.01)	1.59*** (0.02)	1.90*** (0.01)	1.61*** (0.02)	1.90*** (0.01)	1.71*** (0.02)	1.90*** (0.01)	1.78*** (0.02)	1.97*** (0.01)
Observations	9573	9573	9573	9573	9573	9573	9573	9573	9573	9573
Number of iid		3191		3191		3191		3191		3191
R-squared	0.07	0.01	0.08	0.01	0.06	0.01	0.05	0.01	0.08	0.09
Labour shock at wave 1	Yes		Yes		Yes		Yes		Yes	
Labour shock at wave 2–3	Yes		Yes		Yes		Yes		Yes	
Wave Fixed Effects	Yes	Yes	Yes	Yes	Yes	Yes	Yes	Yes	Yes	Yes
Individual Fixed Effects		Yes		Yes		Yes		Yes		Yes

Data from the Longitudinal Study by Bogliacino et al. (2020). Outcomes: Index of mental health and difficulty to isolate. Labour shock is equal to one if the shock occurs after wave I. I[t = k] is a dummy equal to one for observations from wave k. Financial Strain is a dummy equal to one if the participant answered “1 month or less” or “2–3 months” to the question “If you lose your job, for how long do you believe you could pay your bills?”. Bad health is a dummy equal to one if the participant answered “Neither good not bad”, “bad” or “very bad” to the question “How is your health in general?”. Worried is a dummy equal to one if the participant has an index greater than zero. The index is created using a principal component analysis on pessimism over the future, pessimism over the job, fear of a new outbreak, fear of an economic depression, fear of restrictions of rights and freedoms. Mindset is dummy equal to one if the participant had an index greater than zero. The index is created using a principal component analysis on Q7–Q13 in the SOM (Global Preference Survey by Falk et al., 2018)

*OLS* Ordinary Least Squares, *FE* Fixed Effect

Clustered standard errors in parentheses ***p < 0.01, **p < 0.05, *p < 0.1

The tests support the claim that the estimated coefficients are equivalent to zero at the specified threshold. For each coefficient of interest, remember that there are two separate tests to jointly assess that $\left|t\right|\ge \varepsilon$. Thus, we report two p-values, one for $\varepsilon -t\le 0$ and the other for $t+\varepsilon \le 0$. For all cases, we reject the null hypothesis: for the financial strain mediator, p = 0.04 and p = 0.01; for the health mediator, p = 0.00 and p = 0.07; for the worries and uncertainty, p = 0.00 and p = 0.05, and, for the resilience, p = 0.01 and p = 0.03. The only case for which the test is not conclusive is for social isolation, as the null is rejected on one side, p = 0.17 and p = 0.00. We should also stress that the specified threshold is 70% of a standard deviation. This means that our results are compatible with a small to medium size effect.

Finally, we addressed the issue of attrition and external validity, as explained in Sect. 3.3. To estimate the average treatment effect, we need two further assumptions. One assumption is a version of the common support: the probability of receiving a negative shock should be positive and lower than one on the original sample, conditional on the set of variables that we observe in wave I. The other assumption states that attrition is independent of the treatment in waves II and III, conditional on those same variables (gathered in the first wave). Codagnone et al. (2021) estimate the likelihood of survival using a random forest algorithm computed over the full set of health and socio-demographic variables from wave I. We can use the inverse of these probabilities as analytical weights in the regression. The effects reported in Table 5 are approximately 15 to 21% of a standard deviation.

**Table 5:** The effect of negative labour market shock on mental health during the pandemic: Weighted regressions

	(1)	(2)	(3)	(4)
Outcome: stress, anxiety and depression	FE	FE	FE	FE
Labour shock X I[t = 2]		0.11*** (0.04)		
Labour shock X I[t = 3]		0.12*** (0.04)		
Labour shock X I[t = 2,3]	0.13*** (0.03)			
Negative events [+ 1] X I[t = 2,3]			0.11*** (0.02)	
Negative events [+ 1sd] X I[t = 2,3]				0.16*** (0.03)
Constant	1.91*** (0.01)	1.91*** (0.01)	1.91*** (0.01)	1.91*** (0.01)
Observations	9573	9573	9573	9573
Number of iid	3191	3191	3191	3191
R-squared	0.01	0.01	0.01	0.02
Wave fixed effects	Yes	Yes	Yes	Yes
Individual fixed effects	Yes	Yes	Yes	Yes

Data from the Longitudinal Study by Bogliacino et al. (2020). Outcome: index of mental health. Labour shock is equal to one if the shock occurs after wave I. Negative events [+1] is a dummy equal to one if a participant reported at least one more negative event in waves II and III with respect to wave I. Negative events [+1sd] is a dummy equal to one if a participant reported at least one point eight more negative event in waves II and III with respect to wave I. I[t = k] is a dummy equal to one for observations from wave k. Weighted regression, analytical weights are the inverse of the probability of answering the three waves of the survey, estimated using random forest in Codagnone et al. (2021)

*FE* Fixed Effect

Clustered standard errors in parentheses ***p < 0.01, **p < 0.05, *p < 0.1

## Discussion and conclusion

This article used a novel dataset to address the following question: will a negative labour market shock affect stress, anxiety, and depression? This question has gained prominence in recent public and policy discussions because of aggregate adverse events that have been extraordinary, such as the Great Recession and the Covid-19 pandemic. Although notable sociologists and social psychologists have long argued that adverse labour market outcomes are harmful to psychological well-being (Goldsmith et al., 1997), and although the mental health threat has featured prominently in discussions around Covid-19, evidence from the last two decades is still limited in scope.

Following a large and representative sample of individuals in Italy, Spain, and the United Kingdom around the first Covid-19 lockdown (April–May 2020), we were able to track the levels of stress, anxiety and depression, and the change in labour market status. We performed a difference-in-differences analysis to estimate the average treatment effect on the treated exploiting the timing of the shocks within the time window of data collection.

This article has some notable strengths. First, we provided compelling causal evidence on the impact of labour market shocks on mental health: a shock increases mental health problems to a critical level. Second, results are robust to how we measure shocks and control potential heterogeneous effects. Third, we systematically assess potential mediators to determine whether they are at play in the context of the three countries studied during the first wave of Covid-19.

Nonetheless, our study presents limitations. For example, the use of self-reported answers to assess stress, anxiety and depression are said to underestimate the true prevalence (Bharadwaj et al., 2017). Nevertheless, the lockdown, where hospitals and mental health clinics had restricted access, made administrative data inaccessible or unreliable to conduct this type of study. Moreover, self-assessment is a necessary step, even in the clinical diagnosis of mental health.

One could argue that we are not strictly measuring mental health since our index measures the three outcomes along a continuum where there is no threshold to determine when the condition becomes pathological. The critique holds but out method is in line with the Diagnostic and Statistical Manual of Mental Disorders (DSM). In mental health, differently from other subfields, the categorization of mental health pathologies is mostly symptomatic instead of being based on root causes. This has been widely criticized for the case of trauma, in particular developmental trauma (Van Der Kolk, 2014). In this paper we are concerned with forms of toxic stress and anxiety that can lead to several depressive disorders. Our use of the triad stress, anxiety, and depression is motivated by our diagnostic questionnaire and shares the spirit of the DSM. Further studies with a more precise pathological definition would fruitfully complement our results.

Our research design cannot exclude a priori that other types of time varying shocks may affect differentially our sample. Female, low educated, and poor households suffered more severely. Our results are robust: we have staggered treatments which make the cooccurrence of other shocks more unlikely, we control for day-by-country fixed effect, we have some evidence on lack of pre trends, and in one specification we control for dynamic effects of gender, education, and income. Of course, there is an intrinsic limit of this type of research design. More systematic evidence on negative labor market shocks is necessary to inform this research question.

Another limitation is the reduced period of the study. We certainly cannot claim to have identified long run effect, due to the limited time span covered by this study. A follow up would be interesting to assess whether these effects are long run. The limited baseline information on participants partly restricted our ability to assess pre-trends.

Overall, the R-squared across different specifications is quite small. Our main effect explains less than 5% of the variance. Two factors concur to this finding, in our opinion. First, structurally, stress, anxiety and depression are explained by a very large arrays of factors, even over a relatively limited time window. Second, large shocks in the labour market are not that common. Although the negative impact of the pandemic has been remarkable especially during the first wave, for identification, we must rely on those that were hit after the first week.

One final limitation concerns attrition, which is significant in the dataset. We have tried to assess robustness to attrition in terms of internal and external validity, but we should refrain by any conclusion.

Since 2008, all industrialized countries have faced a serious economic recession triggered by the widespread financial crisis in Europe and the United States of America, followed a decade later by a global pandemic, which most researchers and policy makers are aware would likely affect the wellbeing of households, including individuals’ mental wellbeing (WHO, 2011). Experience suggests that crises, such as the Covid-19 pandemic, are pervasive, protracted, and deeply affecting. These findings require a policy effort to soften the negative consequences of major aggregate shocks. Our findings have broad implications for this type of events.

Notably, some groups may be more vulnerable than others to the psychosocial effects of situations such as the Covid-19 pandemic (Pfefferbaum & North, 2020). More specifically, Rajkumar (2020) shows that 16–28% of the subjects screened have reported symptoms of anxiety and depression because of the Covid-19 pandemic, and 8% have self-reported stress. In particular, individuals who have contracted the disease, those at heightened risk (e.g., the elderly, those with compromised immune functions, and those living in healthcare settings of receiving care) and those with pre-existing medical, psychiatric or substance use/abuse issues are at greater risk of adverse psychosocial outcomes (Pan et al., 2020). Mass home-confinement directives (including stay-at-home orders, self-quarantine, and isolation) were new to many citizens living in this current age. Our findings are complementary and suggest that those suffering labour market shocks, which were not immediately targeted by the authorities, may be at serious health risk.

## Data Availability

Data are available upon request.

## Appendix

See Tables 6, 7, 8.

**Table 6:** Descriptive statistics: sample for the first wave compared with the overall population

Panel A						
Share of population 2019 (%)						
	M			F		
	18–25	26–55	56–75	18–25	36–55	56–75
Spain						
North-East	3.3%	5.4%	4.1%	3.3%	5.5%	3.7%
Madrid and Centre	3.4%	5.6%	4.0%	3.5%	5.6%	3.7%
Central East	1.7%	2.8%	2.1%	1.8%	2.9%	1.9%
North	1.0%	2.0%	1.7%	1.1%	1.9%	1.5%
South	3.7%	5.7%	3.8%	3.8%	5.8%	3.6%
Italia						
Central Italy	2.4%	4.2%	3.5%	2.5%	4.1%	3.1%
Islands	1.5%	2.2%	1.9%	1.6%	2.2%	1.7%
North	5.5%	9.5%	8.0%	5.8%	9.5%	7.3%
South	3.2%	4.7%	3.9%	3.4%	4.5%	3.6%
UK						
East of England and Midlands	4.0%	4.7%	4.0%	4.1%	4.6%	3.8%
London	2.7%	2.7%	1.5%	2.7%	2.7%	1.4%
North	3.8%	4.2%	3.7%	3.9%	4.1%	3.5%
South	3.3%	4.2%	3.7%	3.4%	4.0%	3.5%
Scotland and Wales	2.5%	2.9%	2.6%	2.6%	2.8%	2.4%

Panel B						
Share of sample in Wave I (%)						
	M			F		
	18–25	26–55	56–75	18–25	36–55	56–75
Spain						
North-East	3.7%	6.4%	1.8%	3.7%	5.2%	2.0%
Madrid and Centre	5.5%	7.3%	1.3%	5.5%	7.3%	2.1%
Central East	2.4%	4.2%	1.5%	2.8%	4.3%	1.8%
North	2.0%	4.5%	0.9%	2.1%	4.1%	1.6%
South	2.6%	4.0%	0.9%	3.1%	4.3%	1.2%
Italia						
Central Italy	4.2%	5.7%	1.8%	4.4%	4.3%	2.5%
Islands	1.9%	2.7%	0.9%	1.2%	2.3%	1.2%
North	7.7%	11.7%	3.5%	7.0%	9.4%	6.1%
South	4.3%	5.0%	1.3%	3.7%	4.8%	2.4%
UK						
East of England and Midlands	5.5%	7.2%	2.5%	2.8%	5.6%	2.8%
London	5.1%	3.0%	0.8%	5.1%	4.5%	0.9%
North	4.2%	6.8%	2.8%	3.7%	7.3%	2.6%
South	2.8%	3.9%	2.9%	1.8%	3.4%	3.0%
Scotland and Wales	1.1%	1.8%	1.3%	0.9%	2.1%	1.9%

**Table 7:** Multinomial logit analysis of attrition

	(1)	(2)	(3)
Variables	Dropout	Never shocked	Shocked at wave II or III
Female		0.10 (0.07)	− 0.02 (0.05)
Age		0.07*** (0.00)	0.02*** (0.00)
Married		0.08 (0.08)	− 0.08 (0.05)
Education		0.03 (0.03)	0.03 (0.02)
Income Rank		0.14*** (0.04)	0.20*** (0.03)
Unemployed		− 1.25*** (0.16)	− 0.70*** (0.08)
Home ownership		0.08 (0.07)	0.21*** (0.05)
Household size		− 0.07** (0.03)	− 0.02 (0.01)
Urban		− 0.29*** (0.07)	− 0.18*** (0.05)
Kids of school age		− 0.15*** (0.05)	− 0.05** (0.02)
Comorbidity		− 0.05* (0.03)	− 0.01 (0.02)
Constant		− 4.31*** (0.22)	− 1.50*** (0.13)
Observations	8922	8922	8922

Standard errors in parentheses

***p < 0.01, **p < 0.05, *p < 0.1

**Table 8:** Controlling for time variant effect of gender, income, education

	(1)	(2)
Variables	FE	FE
Labor shock X I[t = 2,3]	0.13*** (0.03)	0.13*** (0.03)
Constant	1.90*** (0.01)	1.90*** (0.09)
Observations	9573	9573
R-squared	0.01	0.02
Number of iid	3191	3191
Wave Fixed Effects	Yes	
Fixed effects	Yes	Yes
Time trend for female, income, education	Yes	Yes
Day by country fixed effects		Yes

Clustered standard errors in parentheses

***p < 0.01, **p < 0.05, *p < 0.1

## Footnotes

1 Technically, if the sample or the population mainly relies on employment or self-employment and does not possess any relevant assets, aggregate shocks can be treated as labor market shock. This may hold for studies on developing countries but is unlikely to be the case for developed countries.

2 Although precise, this definition of shocks cannot distinguish between temporary and permanent shock, for which a follow up would be required. We acknowledge this as limitation of the study.

3 [Risk] How do you see yourself: are you a person who is generally willing to take risks, or do you try to avoid taking risks? [Patience] In comparison to others, are you a person who is generally willing to give up something today in order to benefit from that in the future or are you not willing to do so? [Trust] How well does the following statement describe you as a person? As long as I am not convinced otherwise, I assume that people have only the best intentions. [Altruism] Imagine the following situation: you won 1000 euros in a lottery. Considering your current situation, how much would you donate to charity? [Altruism] How do you assess your willingness to share with others without expecting anything in return when it comes to charity? [Positive Reciprocity] 12) imagine the following situation: you are shopping in an unfamiliar city and realize you lost your way. You ask a stranger for directions. The stranger offers to take you with their car to your destination. The ride takes about 20 min and costs the stranger about 20 euros in total. The stranger does not want money for it. You carry six bottles of wine with you. The cheapest bottle costs 5 euros, the most expensive one 30 euros. You decide to give one of the bottles to the stranger as a thank-you gift. Which bottle do you give? [Negative Reciprocity] How do you see yourself: Are you a person who is generally willing to punish unfair behaviour even if this is costly?

## References

[CR1] Adams-Prassl A, Boneva T, Golin M, Rauh C (2022). The impact of the coronavirus lockdown on mental health: Evidence from the United States. Economic Policy.

[CR2] Altindag O, Erten B, Keskin P (2022). Mental health costs of lockdowns: evidence from age-specific curfews in Turkey. American Economic Journal: Applied Economics.

[CR3] Angeles G, de Hoop J, Handa S, Kilburn K, Milazzo A, Peterman A (2019). Government of Malawi’s unconditional cash transfer improves youth mental health. Social Science and Medicine.

[CR4] Baird S, de Hoop J, Özler B (2013). Income shocks and adolescent mental health. Journal of Human Resources.

[CR5] Baker AC, Larcker DF, Wang CCY (2022). How much should we trust staggered difference-in-differences estimates?. Journal of Financial Economics.

[CR6] Baranov V, Grosjean P, Khan FJ, Walker S (2022). The impact of COVID-related economic shocks on household mental health in Pakistan. Health Economics.

[CR7] Barr B, Kindeman P, Whitehead M (2015). Trends in mental health inequalities in England during a period of. Social Science and Medicine.

[CR8] Bauer M (2016). Can war foster cooperation?. Journal of Economic Perspectives.

[CR9] Beck AT, Alford BA (2009). Depression causes and treatment.

[CR10] Beck A, Beamesderfer A, Pichot P, Olivier-Martin R (1974). Assessment of depression: The depression inventory. Psychological measurements in psychopharmacology.

[CR11] Belot MS, Choi ET, van den Broek-Altenburg E, Jamison JC, Papageorge NW (2021). Unequal consequences of Covid19 across age and income: Representative evidence from six countries. Review of Economics of the Household, Volumen.

[CR13] Bharadwaj P, Pai M, Suziedelyte A (2017). mental health stigma. Economics Letters.

[CR14] Bianchi F, Bianchi G, Song D (2020). The long-term impact of the COVID-19 unemployment shock on life expectancy and mortality rates. Journal of Economic Dynamics and Control.

[CR15] Bogliacino, F. et al. (2020). Longitudinal study on behavioral, psychological, socio-economic effects induced by the exposure to CoViD-19, by government measures, and by communication strategies in three countries. Italy, Spain, and United Kingdom.

[CR16] Bogliacino F, Codagnone C, Montealegre F, Folkvord F, Gómez C, Charris R, Liva G, Lupiáñez-Villanueva F, Veltri GA (2021). Negative shocks predict change in cognitive function and preferences: Assessing the negative affect and stress hypothesis. Scientific Reports.

[CR17] Bogliacino F, Grimalda G, Ortoleva P, Ring P (2017). Exposure to and recall of violence reduce short-term memory and cognitive control. Proceedings of the National Academy of Sciences.

[CR18] Bogliacino F, Montealegre A (2020). Do negative economic shocks affect cognitive function, adherence to social norms and loss aversion?. Journal of the Economic Science Association.

[CR19] Bonanno GA, Galea S, Bucciarelli A, Vlahov D (2007). What predicts psychological resilience after disaster? The role of demographics, resources, and life stress. Journal of Consulting and Clinical Psychology.

[CR20] Branas CC (2015). The impact of economic austerity and prosperity events on suicide in Greece: A 30-year interrupted time-series analysis. British Medical Journal Open.

[CR21] Brand JE (2015). The far-reaching impact of job loss and unemployment. Annual Review of Sociology.

[CR22] Bromet E (2011). Cross-national epidemiology of DSM-IV major depressive episode. BMC Medicine.

[CR23] Brooks S (2020). The psychological impact of quarantine and how to reduce it: Rapid review of the evidence. Lancet.

[CR26] Callaway B, Sant'Anna PH (2021). Difference-in-differences with multiple time periods and an application on the minimum wage and employment. Journal of Econometrics.

[CR27] Cardeña E (2000). Psychometric properties of the stanford acute stress reaction questionnaire (SASRQ): A valid and reliable measure of acute stress. Journal of Traumatic Stress.

[CR28] Case A, Deaton A (2015). Rising morbidity and mortality in midlife among white. Proceedings of the National Academy of Sciences.

[CR29] Case A, Deaton A (2020). Deaths of despair and the future of Capitalism.

[CR30] Cengiz D, Dube A, Lindner A, Zipperer B (2019). The effect of minimum wages on low-wage jobs*. The Quarterly Journal of Economics.

[CR31] Chang S-S, Gunnell D, Sterne JAC, Lu T-H, Cheng ATA (2009). Was the economic crisis 1997–1998 responsible for rising suicide rates in East/Southeast Asia? A time–trend analysis for Japan, Hong Kong, South Korea, Taiwan, Singapore and Thailand. Social Science & Medicine.

[CR33] Clark AE (2003). Unemployment as a social norm: Psychological evidence from panel data. Journal of Labor Economics.

[CR34] Clark AE, Georgellis Y, Sanfey P (2001). Scarring: The psychological impact of past unemployment. Economica.

[CR35] Codagnone C, Bogliacino F, Gómez C, Charris R, Montealegre F, Liva G, Folkvord F, Lupiáñez-Villanueva F, Veltri GA (2020). Assessing concerns for the economic consequence of the COVID-19 response and mental health problems associated with economic vulnerability and negative economic shock in Italy, Spain, and the United Kingdom. PLoS ONE.

[CR36] Codagnone C, Bogliacino F, Gómez C, Folkvord F, Liva G, Charris R, Montealegre F, Lupiáñez-Villanueva F, Veltri GA (2021). Restarting “Normal” Life after Covid-19 and the lockdown: Evidence from Spain, the United Kingdom, and Italy. Social Indicators Research.

[CR37] Cole K, Daly A, Mak A (2009). Good for the soul: The relationship between work, wellbeing and psychological capital. The Journal of Socio-Economics.

[CR38] Connor K, Zhang W (2006). Resilience: determinants, measurement, and treatment responsiveness. CNS Spectrums.

[CR40] Cutler DM, Lleras-Muney A, Vogl T, Glied S, Smith PC (2012). Socioeconomic status and health: Dimensions and mechanisms'. The Oxford handbook of health economics.

[CR41] Cygan-Rehm K, Kuehnle D, Oberfichtner M (2017). Bounding the causal effect of unemployment on mental health: nonparametric evidence from four countries. Health Economics..

[CR42] de Quidt J, Haushofer J (2019). Depression through the lens of economics. The economics of poverty traps.

[CR44] Durkheim É (1951). Suicide: a study in sociology.

[CR45] Economou M (2013). Major depression in the Era of economic crisis: A replication. Journal of Affective Disorders.

[CR46] Etheridge, B. & Spantig, L. (2020). The gender gap in mental well being during the Covid19 outbreak: evidence from the UK. ISER Working paper series.

[CR47] Ettman CK, Abdalla SM, Cohen GH, Sampson L, Vivier PM, Galea S (2020). Prevalence of depression symptoms in US adults before and during the COVID-19 pandemic. JAMA Network Open.

[CR48] Eyal K, Burns J (2019). The parent trap: Cash transfers and the intergenerational transmission of depressive symptoms in South Africa. World Development.

[CR50] Falk A (2018). Global evidence on economic preferences. Quarterly Journal of Economics.

[CR51] Flatau P, Galea J, Petridis R (2000). Mental health and wellbeing and unemployment. Australian Economic Review.

[CR52] Gili M (2012). The mental health risks of economic crisis in Spain: evidence from primary care centres, 2006 and 2010. European Journal of Public Health.

[CR53] Goldsmith AH, Veum JR, Darity WJ (1997). Unemployment, Joblessness, psychological well-being and self esteem: theory and evidence. The Journal of Socio-Economics.

[CR55] Gruber J (1994). The incidence of mandated maternity benefits. American Economic Review.

[CR56] Gualano MR, Lo Moro G, Voglino G, Bert F, Siliquini R (2020). Effects of Covid-19 lockdown on mental health and sleep disturbances in Italy. International Journal of Environmental Research and Public Health.

[CR57] Hampshire A, Hellyer PJ, Soreq E, Mehta MA, Ioannidis K, Trender W, Chamberlain SR (2021). Associations between dimensions of behaviour, personality traits, and mental-health during the COVID-19 pandemic in the United Kingdom. Nature Communications.

[CR58] Haushofer J, Chemin M, Jang C, Abraham J (2020). Economic and psychological effects of health insurance and cash transfers: Evidence from a randomized experiment in Kenya. Journal of Development Economics.

[CR59] Haushofer J, Fehr E (2014). On the psychology of poverty. Science.

[CR60] Haushofer J, Shapiro J (2016). The short-term impact of unconditional cash transfers to the poor: Experimental evidence from Kenya*. The Quarterly Journal of Economics.

[CR61] Heckman JJ (2010). Building bridges between structural and program evaluation approaches to evaluating policy. Journal of Economic Literature.

[CR62] Hensel L, Witte M, Caria AS, Fetzer T, Fiorin S, Götz FM, Gomez M, Haushofer J, Ivchenko A, Kraft-Todd G, Reutskaja E, Roth C, Yoeli E, Jachimowicz JM (2022). Global behaviors, perceptions, and the emergence of social norms at the onset of the COVID-19 pandemic. Journal of Economic Behavior & Organization.

[CR63] Hoang TTA, Knabe A (2021). Time use, unemployment, and well-being: An empirical analysis using British time-use data. Journal of Happiness Studies.

[CR65] Hong SH (2011). Measuring the effect of napster on recorded music sales: Difference-in-differences estimates under compositional changes. Journal of Applied Econometrics.

[CR66] Jahoda M (1981). Work, employment, and unemployment: Values, theories, and approaches in social research. American Psychologist.

[CR67] Jahoda M (1982). Employment and unemployment. A social-psychological analysis.

[CR68] Jahoda M, Lazarsfeld P, Zeisel H (1971). Marienthal—The sociography of an unemployed community.

[CR69] Jia R, Ayling K, Chalder T, Massey A, Broadbent E, Coupland C, Vedhara K (2020). Mental health in the UK during the COVID-19 pandemic: cross-sectional analyses from a community cohort study. British Medical Journal Open.

[CR70] Kilburn K, Handa S, Angeles G, Tsoka M, Mvula P (2018). Paying for happiness: Experimental results from a large cash transfer program in Malawi. Journal of Policy Analysis and Management.

[CR71] Kozlowski, J., Veldkamp, L. & Venkateswaran, V. (2020). Scarring body and mind: The long-term belief-scarring effects of COVID-19, Federal Reserve Bank of St Louis working paper 2020–009A

[CR72] Lakens D (2017). Equivalence tests: A practical primer for t tests, correlations, and meta-analyses. Social Psychological and Personality Science.

[CR73] Laudenbach C, Malmendier U, Niessen-Ruenzi A (2019). Emotional tagging and belief formation: The long-lasting effects of experiencing communism. AEA Papers and Proceedings.

[CR74] Loades ME, Chatburn E, Higson-Sweeney N, Reynolds S, Shafran R, Brigden A, Crawley E (2020). Rapid systematic review: The impact of social isolation and loneliness on the mental health of children and adolescents in the context of COVID-19. Journal of the American Academy of Child & Adolescent Psychiatry.

[CR75] Lovibond SH, Lovibond PF (1995). Manual for the depression anxiety & stress scales.

[CR76] Lowe S, Galea S (2017). The mental health consequences of mass shootings. Trauma, Violence, & Abuse.

[CR77] Lund C (2010). Poverty and common mental disorders in low and middle income countries: A systematic review. Social Science and Medicine.

[CR78] Lund C (2011). Poverty and mental disorders: Breaking the cycle in low-income and middle-income countries. The Lancet.

[CR79] Luthar SS, Zelazo LB, Luthar SS (2012). Research on resilience: An integrative review. Resilience and vulnerability.

[CR80] Malmendier, U. & Sheng Shen, L. (2019). Scarred Consumption. International Finance Discussion Papers, Issue 1259.

[CR81] Malmendier U (2021). Experience effects in finance: foundations, applications, and future directions. Review of Finance.

[CR82] Malmendier U (2021). Why personal histories matter in in economics. Journal of the European Economic Association.

[CR83] Malmendier U, Nagel S (2011). Depression babies: Do macroeconomic experiences affect risk taking?. Quarterly Journal of Economics.

[CR84] Mani A, Mullainathan S, Shafir E, Zhao J (2013). Poverty impedes cognitive function. Science.

[CR85] McInerney M, Mellor JM, Hersch Nicholas L (2013). Recession depression: Mental health effects of the 2008 stock market crash. Journal of Health Economics.

[CR86] McIntyre RS, Lee Y (2020). Preventing suicide in the context of the COVID-19 pandemic. World Psychiatry.

[CR87] McIntyre RS, Lee Y (2020). Projected increases in suicide in Canada as a consequence of COVID-19. Psychiatry Research.

[CR88] McKee-Ryan F, Song Z, Wanberg CR, Kinicki AJ (2005). Psychological and physical well-being during unemployment: A meta-analytic study. Journal of Applied Psychology.

[CR89] Merton R (1938). Social structure and anomie. American Sociological Review.

[CR90] Milner A, Page A, LaMontagne AD (2013). Long-term unemployment and suicide: A systematic review and meta-analysis. PLoS ONE.

[CR92] Navarro-Carrillo G, Alonso-Ferres M, Moya M, Valor-Segura I (2020). Socioeconomic status and psychological well-being: Revisiting the role of subjective socioeconomic status. Frontiers in Psychology.

[CR93] Niedzwiedz CL, Green MJ, Benzeval M, Campbell D, Craig P, Demou E, Katikireddi SV (2021). Mental health and health behaviours before and during the initial phase of the COVID-19 lockdown: Longitudinal analyses of the UK Household Longitudinal Study. Journal of Epidemiology and Community Health.

[CR94] Nochaiwong S, Ruengorn C, Thavorn K, Hutton B, Awiphan R, Phosuya C, Wongpakaran T (2021). Global prevalence of mental health issues among the general population during the coronavirus disease-2019 pandemic: A systematic review and meta-analysis. Scientific Reports.

[CR95] Ogburn WF, Thomas DS (1922). The influence of the business cycle on certain social conditions. Journal of the American Statistical Association.

[CR97] Pan K-Y (2020). The mental health impact of the COVID-19 pandemic on people with and without depressive, anxiety, or obsessive-compulsive disorders: A longitudinal study of three Dutch case-control cohorts. Lancet Psychiatry.

[CR98] Pfefferbaum B, North CS (2020). Mental health and the Covid-19 pandemic. New England Journal Fo Medicine.

[CR100] Price RH, Choi JN, Vinokur AD (2002). Links in the chain of adversity following job loss: How financial strain and loss of personal control lead to depression, impaired functioning, and poor health. Journal of Occupational Health Psychology.

[CR101] Rajkumar R (2020). COVID-19 and mental health: A review of the existing literature. Asian Journal of Psychiatry.

[CR102] Reeves A, McKee M, Stuckler D (2014). Economic suicides in the great recession in Europe and North America. British Journal of Psychiatry.

[CR103] Reeves A, Stuckler D, McKee M, Gunnell D, Chang S-S, Basu S (2012). Increase in state suicide rates in the USA during economic recession. The Lancet.

[CR104] Ridley M, Rao G, Schilbach F, Patel V (2020). Poverty, depression, and anxiety: Causal evidence and mechanisms. Science.

[CR107] Rossin-Slater M, Schnell M, Schwandt H, Trejo S, Uniat L (2020). Local exposure to school shootings and youth antidepressant use. Proceedings of the National Academy of Sciences.

[CR108] Ruhm CJ (2000). Are recessions good for your health?. Quarterly Journal of Economics.

[CR111] Seligman MEP (1975). Helplessness: On depression, development and death.

[CR112] Serafini G, Parmigiani B, Amerio A, Aguglia A, Sher L, Amore M (2020). The psychological impact of COVID-19 on the mental health in the general population. QJM: an International Journal of Medicine.

[CR115] Stuckler D, Basu S, Suhrcke M, Coutts A, McKee M (2009). The public health effect of economic crises and alternative policy responses in Europe: An empirical analysis. The Lancet.

[CR116] Tan JJX, Kraus MW, Carpenter NC, Adler NE (2020). The association between objective and subjective socioeconomic status and subjective well-being: A meta-analytic review. Psychological Bulletin.

[CR117] Thern E, de Munter J, Hemmingsson T, Rasmussen F (2017). Long-term effects of youth unemployment on mental health: Does an economic crisis make a difference?. Journal of Epidemiology and Community Health.

[CR118] Vai B, Mazza MG, Delli Colli C, Foiselle M, Allen B, Benedetti F, De Picker LJ (2021). Mental disorders and risk of COVID-19-related mortality, hospitalisation, and intensive care unit admission: A systematic review and meta-analysis. The Lancet Psychiatry.

[CR1000] Van Der Kolk, B. A. (2014). The body keeps the score. Penguin Random House.

[CR121] Wanberg CR (2011). The individual experience of unemployment. Annual Review of Psychology.

[CR122] Wang Y, Fattore G (2020). The impact of the great economic crisis on mental health care in Italy. The European Journal of Health Economics.

[CR123] WHO. (2011). Impact of economic crises on mental health, s.l.: s.n.

[CR124] Winkelmann R (2009). Unemployment, social capital, and subjective well-being. Journal of Happiness Studies.

[CR125] Young C (2012). Losing a job: The nonpecuniary cost of unemployment in the United States. Social Forces.

